# Inside Out: Modern Imaging Techniques to Reveal Animal Anatomy

**DOI:** 10.1371/journal.pone.0017879

**Published:** 2011-03-22

**Authors:** Henrik Lauridsen, Kasper Hansen, Tobias Wang, Peter Agger, Jonas L. Andersen, Peter S. Knudsen, Anne S. Rasmussen, Lars Uhrenholt, Michael Pedersen

**Affiliations:** 1 MR Research Center, Aarhus University Hospital Skejby, Aarhus, Denmark; 2 Zoophysiology, Department of Biological Sciences, Aarhus University, Aarhus, Denmark; 3 Institute of Clinical Medicine, Aarhus University Hospital Skejby, Aarhus, Denmark; 4 Section for Forensic Pathology, Department of Forensic Medicine, Aarhus University, Aarhus, Denmark; Genentech, United States of America

## Abstract

Animal anatomy has traditionally relied on detailed dissections to produce anatomical illustrations, but modern imaging modalities, such as MRI and CT, now represent an enormous resource that allows for fast non-invasive visualizations of animal anatomy in living animals. These modalities also allow for creation of three-dimensional representations that can be of considerable value in the dissemination of anatomical studies. In this methodological review, we present our experiences using MRI, CT and μCT to create advanced representation of animal anatomy, including bones, inner organs and blood vessels in a variety of animals, including fish, amphibians, reptiles, mammals, and spiders. The images have a similar quality to most traditional anatomical drawings and are presented together with interactive movies of the anatomical structures, where the object can be viewed from different angles. Given that clinical scanners found in the majority of larger hospitals are fully suitable for these purposes, we encourage biologists to take advantage of these imaging techniques in creation of three-dimensional graphical representations of internal structures.

## Introduction

The internal structures of the animal body can be difficult to visualise, and classic dissections require the unique combination of a researcher who is skilful, patient and endowed with appropriate artistic skills. Today, however, three-dimensional computer modelling can provide morphological and anatomical information in a minimal-invasive and much faster fashion [1]. Magnetic resonance imaging (MRI) and computed tomography (CT) are two modalities routinely used to scan cross-sectionally for various diseases in humans, and smaller versions are now manufactured for dedicated animal research. These techniques can image the entire body with little distortion [2], [3]. In addition to static images or movies, computer graphics allow for creation of naturalistic interactive three-dimensional models of the anatomy, where dynamic processes can be simulated and visualised, enabling the researcher to extract skeleton, organs, vascular structures, etc. While both MR and CT have been used extensively to describe human anatomy [4], [5] and the anatomy of classic experimental animal models in experimental medicine (e.g. mouse, rat, rabbit, pig), these techniques have been employed much less in the realm of comparative anatomy [6]–[17]. Amphibian and reptilian anatomy, for example, is today mostly presented in the form of illustrative drawings and pictures [18]–[20]. High-field MRI and micro-CT (μCT) have, nevertheless, been used to characterise the internal morphology of polychaetes [21] and echinoderms [22]–[24]. Paleontologists have used μCT to study fossil invertebrates preserved in amber [25], and high-resolution CT images have been used to compliment molecular data in the construction of phylogenetic relationships amongst squamate reptiles [26]. The web-based library www.digimorph.org hosts a comprehensive collection of three-dimensional representation of animal morphology based on high-resolution CT imaging with particular focus on cranial morphology [27]. In addition, the Digital Fish Library www.digitalfishlibrary.org describes the morphological diversity of fishes using MRI [28].

In the present study, we selected animals from various Classes to illustrate the broad applicability of MR and CT to produce three-dimensional representations of animal anatomy. We demonstrate that MRI and CT are useful techniques to create exact three-dimensional representations (static models, movies and interactive presentations) of various soft and hard tissues and vascular anatomy in various animals.

## Methods

### Animals

Animal procedures were conducted in accordance with the guidelines of the European Communities, Directive 86/609/EEC regulating animal research and approved by the national ethical committee (approval: #2006/561-1192). The animals used were obtained from commercial dealers or imported directly to the Department of Biological Sciences (Aarhus University), where they were maintained for physiological studies. The animals used in this study are presented in Table 1, including anaesthetics used.

**Table 1:** List of animals undergoing CT, μCT, and MRI scanning and the use of anaesthetics.

Animal	Anaesthesia	Image modality	Contrast enhancement
Whiteknee tarantula (*Acanthoscurria geniculate*)	Anaesthesia with 100% CO_2_	MRI	Gastrointestinal structures enhanced by ingestion of Dotarem-filled cockroach
Rice field eel (*Monopterus albus*)	Anaesthesia and termination with 3 g/kg ethyl p-aminobenzoat in water	μCT	Vascular filling with Microfil
African lungfish (*Protopterus annectens*)	Anaesthesia with 3 g/kg ethyl p-aminobenzoat in water	CT	No contrast enhancement applied
Cane toad (*Rhinella marina*)	Anaesthesia and termination with 1% ethyl p-aminobenzoat in water	CT	Vascular filling with Mixobar in gelatinous solution
Monitor lizard (*Varanus exanthematicus*)	Termination with 100 mg/kg pentobarbital	CT	No contrast enhancement applied
American alligator (*Alligator mississippiensis*)	Termination with 100 mg/kg pentobarbital	CT	Vascular filling with Mixobar in gelatinous solution
Ball python (*Python regius*)	Termination with 100 mg/kg pentobarbital	CT	No contrast enhancement applied
Yellow anaconda (*Eunectes notaeus*)	Termination with 100 mg/kg pentobarbital	CT and μCT	Vascular filling with Mixobar in gelatinous solution
Red-eared slider (*Trachemys scripta*)	Termination with 100 mg/kg pentobarbital	CT and MRI	Vascular filling with Mixobar in gelatinous solution
Domestic pig (*Sus scrofa domesticus*)	Premedication with ketamin and midazolam followed by ventilation with 1% isoflurane and termination with pentobarbital	CT	Vascular filling with Mixobar in gelatinous solution
Giraffe (*Giraffa camelopardalis*)	Heart delivered perfusion fixed from an expedition to Africa	CT	Vascular filling with Mixobar in gelatinous solution

For *ex vivo* angiography, the vascular beds of the rice field eel were perfused with a commercially available CT contrast agent (Microfil; Flow Tech Inc., Carver, MA). Vascular fillings of cane toad, anaconda, red-eared slider, pig, and giraffe heart were accomplished with a mixture of gelatine and MRI (Dotarem; Guerbet, Paris, France) and CT (Mixobar Colon; Astra Tech, Mölndal, Sweden) contrast agent as described by Rasmussen *et* al. [7]. Arterial and venous injections were performed on deeply anesthetised animals, terminated in anaesthesia, with a pressure-controlled pump after initial flushing with heparinised saline. Physiological pressure was used as the intentional injection pressure. After filling of the entire vasculature, the animal was cooled on ice to rapidly solidify the injected contrast agents.

### Computed Tomography (CT)

CT provides radiography-based thin cross sectional images without disturbing internal structures. The Röntgen tube, detectors and associated electronics are contained within the gantry that rotates around the animal, which is positioned in the centre of the scanner. During CT scanning, electromagnetic radiation (x-rays) penetrates the study object from 360°. Because some radiation is absorbed by the tissues, the initial data processed by the CT computer system is in fact shadow projections of the absorption from various angles. The pre-defined field of view is divided into volume elements (voxels) and the absorption coefficients, measured in Houndsfield units (HU), of these voxels are computed into elements (pixels), creating thin cross-sectional images of the study object that are displayed as individual grey shades on a screen. These grey shades finally allow differentiation between tissues and structures according to their radiodensity.

In this study, CT scans were performed using a 64-slice Siemens Somatom Definition (Siemens Medical Solutions, Germany). Acquisition parameters included a slice collimation of 4 mm; a pitch of 2°; 32 rotations, a spatial resolution of 0.216 mm^3^/voxel (reconstructed to 0.027 mm^3^/voxel) and a scan duration of 10–30 s (depending on the size of the object). Additional μCT scans were performed using a Scanco Medical μCT 40 scanner (Scanco Medical, Zürich, Switzerland) containing an x-ray source at 80 kV and 160 µA. The μCT images were reconstructed three-dimensionally with an isotropic voxel size of 64×10^6^ µm^3^/voxel.

### Magnetic resonance Imaging (MRI)

MRI provides detailed images of the body in any directional plane by aligning the spin of hydrogen nuclei in the study object by aid of a strong external magnetic field. The study object is divided into voxels by coding field strength, spin frequency and spin phase in three-dimensional space. Hydrogen nuclei situated in various environments, i.e. tissue types, differ by intrinsic magnetic relaxation times, and this physical characteristic is used to produce contrast between different soft tissues. The ability to translate these magnetic relaxation times to grey-white signal intensities makes MRI particularly sensitive to delineate all anatomical structures other than air-filled regions and calcified compounds (skeleton and bones) [29]. In this study, MRI was performed with two clinically available 1.5 Tesla systems (Philips Medical Systems, Netherlands; and Siemens Medical Solutions, Germany). The animal was positioned in a quadrature radiofrequency receiver-coil. A fast localizer scan was followed by a high-resolution 3D gradient–echo sequence with the following parameters: field-of-view depending on animal size; thickness 0.5 mm; TR 23.1 ms; TE 1.6 ms and excitation flip angle 30°. A stack of multiple slices (with no gaps) was acquired, covering the entire specimen of interest with scan durations of 10–60 min. Images were isotropically acquired with a spatial resolution of 0.125 mm^3^/voxel.

### Image analysis

Data acquired both by MRI and CT were exported in DICOM format and 3D reconstructions were generated using the free DICOM-viewer OsiriX software (www.osirix-viewer.com) for Macintosh computers. In OsiriX, DICOM-files were developed as both still-images and animations, and more advanced features were applied: segmentation for highlighting of individual structures, representations of objects as 3D-rotations, determination of flythrough routes, and cropping (in multiple depths, planes, and directions). Quantitative measures (angles, lengths, and volumes) can easily be extracted from the digital models, although we have not included those in this study. Images were exported as tagged image file format (TIFF) in 300 dpi. Movies were exported in QuickTime file format (MOV). As no general agreement exists about the visual enhancement of zoological structures, the computed images and movies were adjusted with regards to brightness, contrast and colour according to personal experiences.

## Results

**Figure 1:**
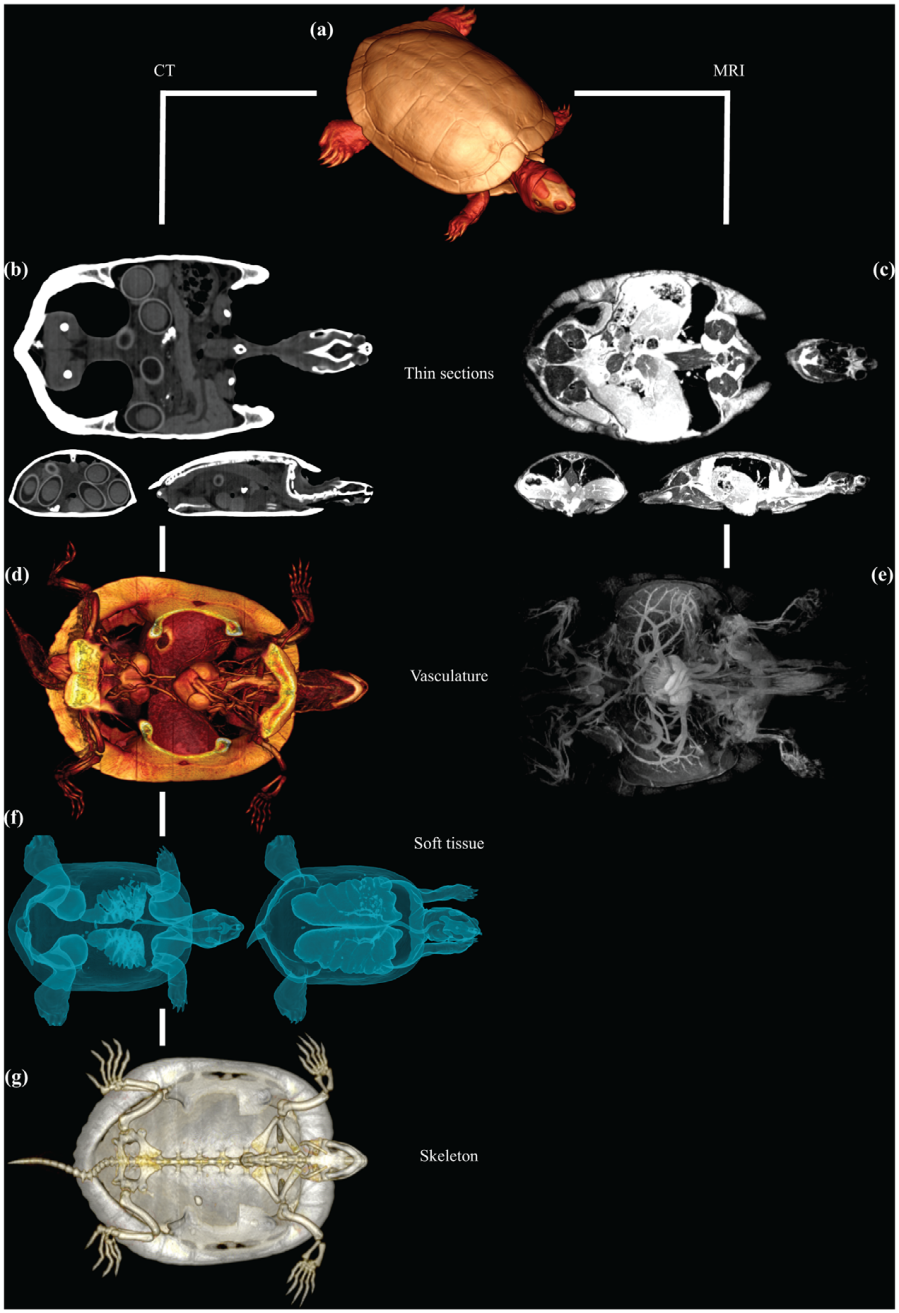
CT and MRI of a red-eared slider. CT (a, b, d, f, g) and MRI (c, e) in red-eared slider (*Trachemys scripta*). CT and MRI have different capacities in visualising vasculature (d, e), soft tissue (c, f) and skeleton (a, g). (**b, c**): Both of the scanning modalities produce thin cross sectional images of the red-eared slider under study. (**a, d, e, f, g**): Further processing of the thin cross sectional images leads to a three dimensional digital model of the animal by the aid of volume rendering software.

**Figure 2:**
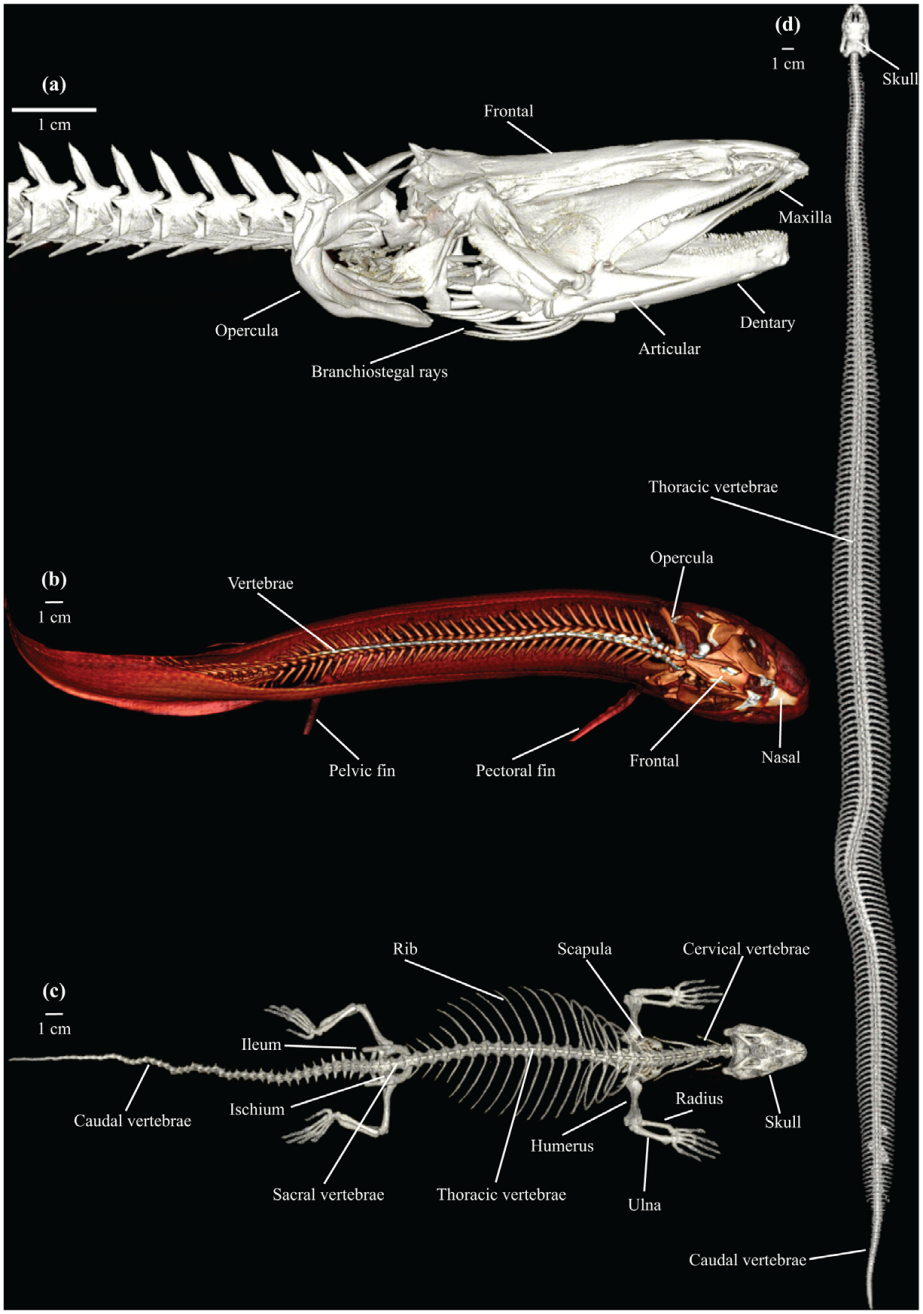
μCT and CT representations of skeletal anatomy. μCT (a) and CT (b, c, d) representations of skeletal anatomy. (**a**): Lateral view of Vietnamese rice field eel (*Monopterus albus*). (**b**): Dorsal view of African lungfish (*Protopterus annectens*). (**c**): Dorsal view of African Savannah monitor (*Varanus exanthematicus*). (**d**): Dorsal view of ball python (*Python regius*).

**Figure 3:**
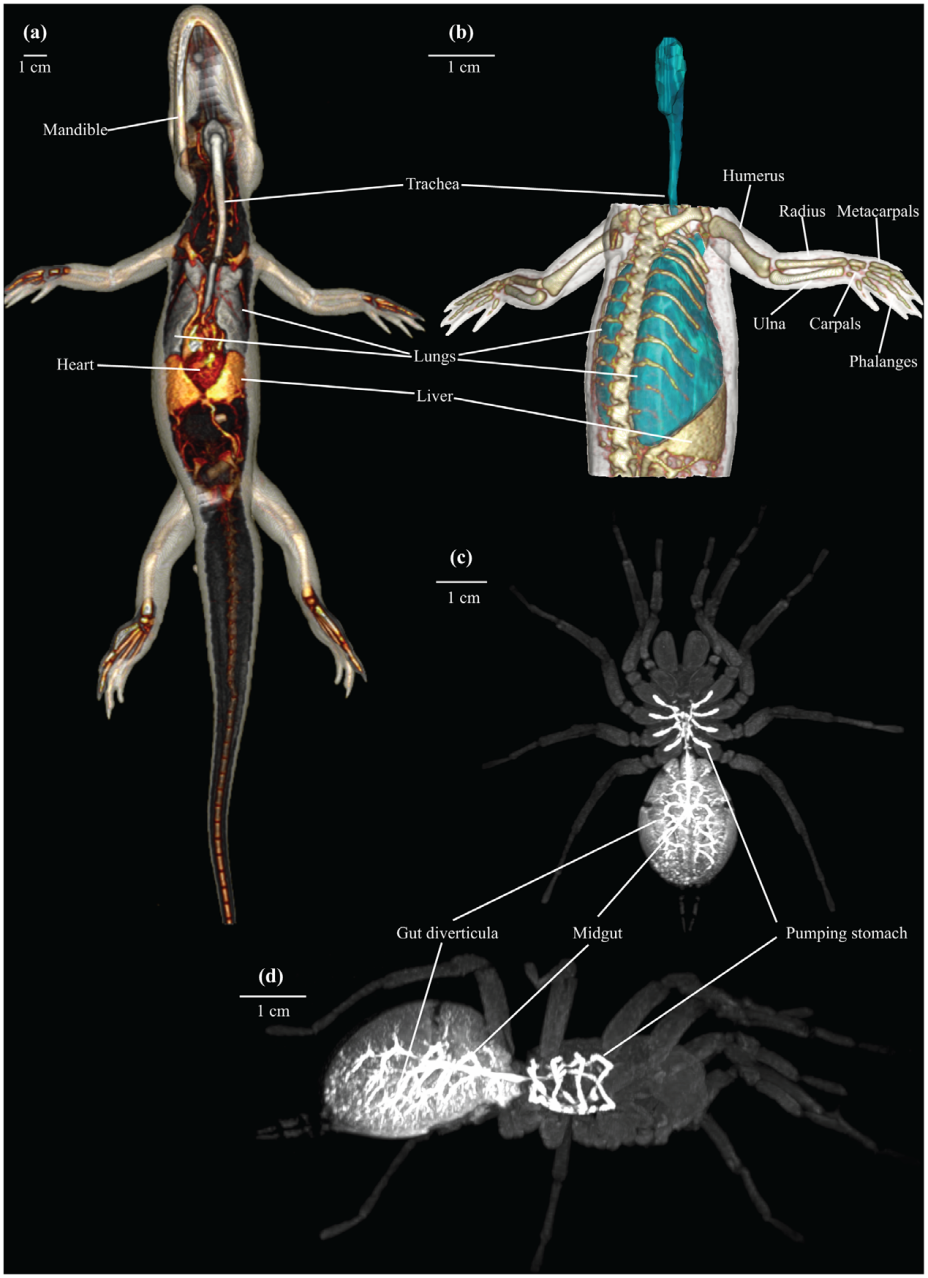
CT and MRI of organ structures. CT (a, b) and MRI (c, d) representations of organ structures. (**a**): Ventral view of American alligator (*Alligator mississippiensis*) with lungs (gray), liver (yellow) and heart (red) highlighted. (**b**): Ventrolateral view of American alligator (*Alligator mississippiensis*) with air-filled structures (lungs and trachea) highlighted blue. (**c**) and (**d**): Coronal (c) and lateral (d) view of whiteknee tarantula (*Acanthoscurria geniculate*) with the gastrointestinal tract enhanced following ingestion of MRI contrast agent.

**Figure 4:**
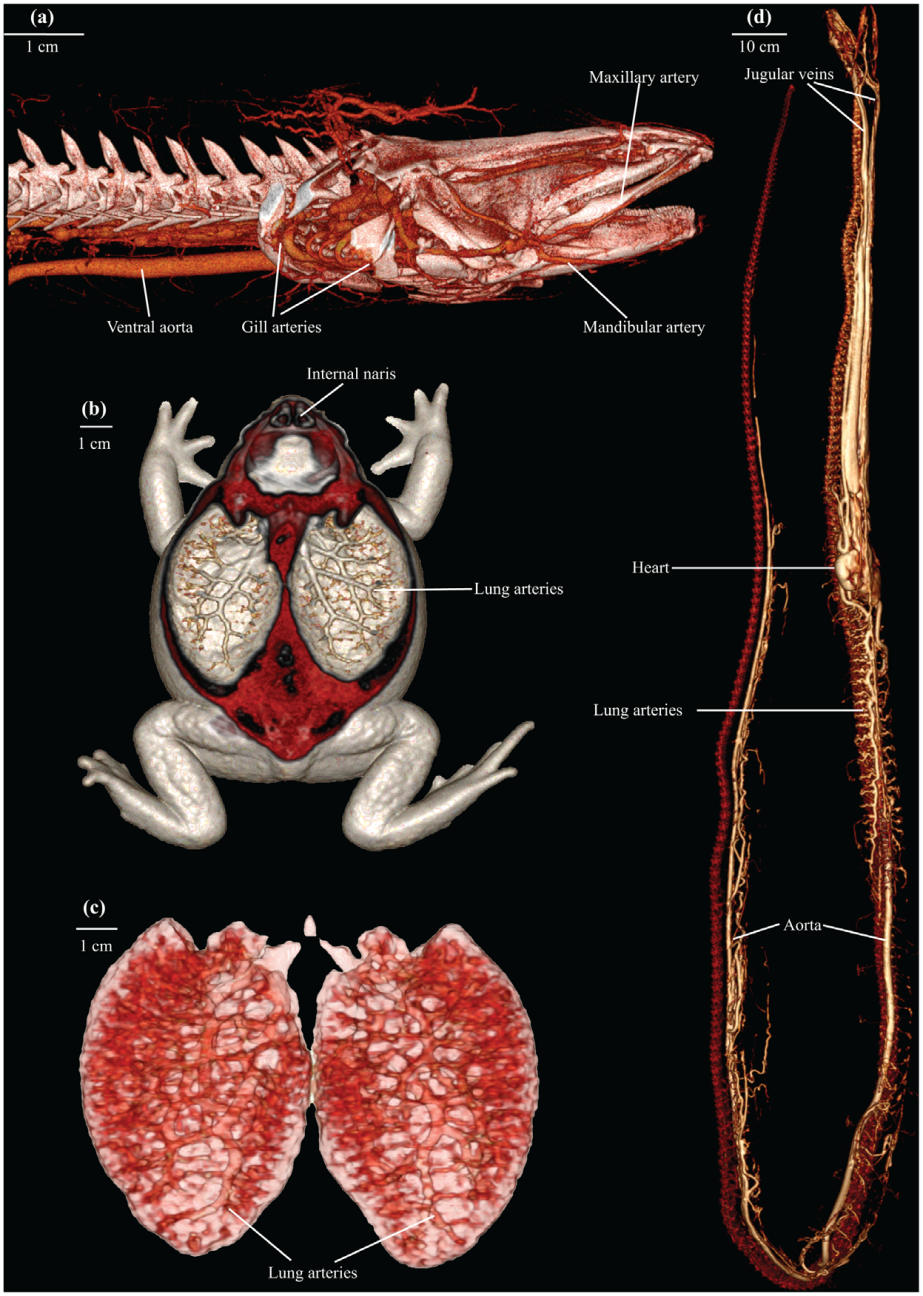
μCT and CT of contrast agent filled vasculatures. μCT (a) and CT (b, c, e) representations of contrast agent filled vasculature. (**a**): Ventral view of the head region of a Vietnamese rice field eel (*Monopterus albus*) with contrast agent filled vascular beds. (**b**): Ventral view of South American cane toad (*Rhinella marina*) with lung arteries outlined. (**c**): Lungs of the South American cane toad (*Rhinella marina*) digitally isolated. (**d**): Ventrolateral view of the vasculature in a yellow anaconda (*Eunectes notaeus*).

**Figure 5:**
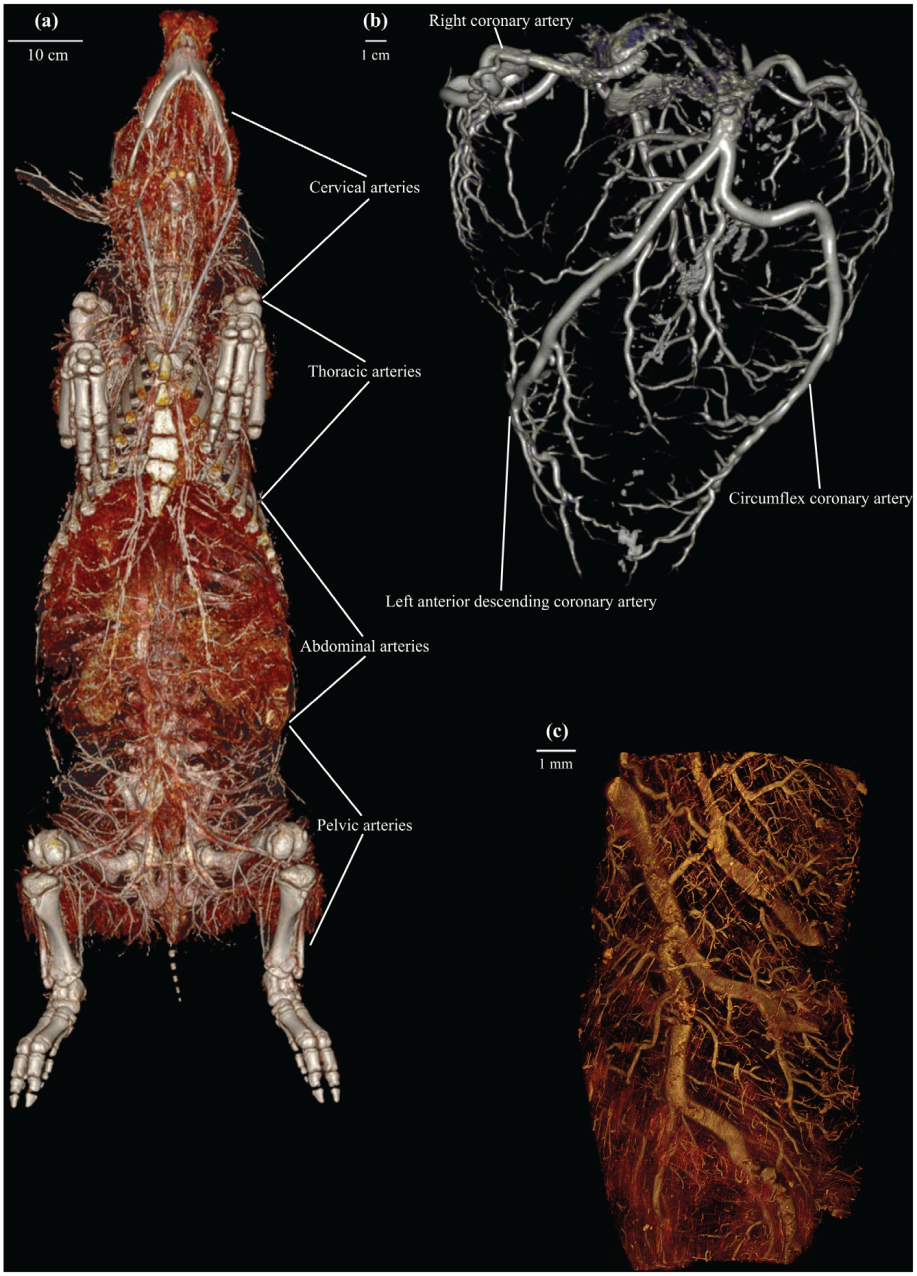
μCT and CT representations of mammalian and reptilian vasculatures. CT (a, b) and μCT (c) representations of contrast filled vasculature in mammals and reptiles. (**a**): Ventral view of the whole body vasculature in a domestic pig (*Sus scrofa domesticus*). (**b**): Coronary arteriography of a giraffe (*Giraffa camelopardalis*) heart. (**c**): Coronary arteries visualised in the heart of a yellow anaconda (*Eunectes notaeus*).

Images of a turtle using MRI and CT imaging, respectively, are shown in Figure 1a–g to highlight the differences between these two modalities. As shown in Figure 1c, MRI allows for detailed visualisation of the various organs at a thin sectional level, whereas CT is particularly useful for harder structures (note for example in Figure 1b the intraabdominal eggs in the thin section with CT) and for radio-density contrasts such as the difference between tissue and air, enabling the lungs to be visualised clearly (Figure 1f). Both MRI and CT allow for visualisations of the vasculature (Figure 1d,1e), however it must be stressed that the vessel and organ structures in the CT image (Figure 1d) are visible only because a CT contrast agent has been injected into the vasculature. Also, an MRI contrast agent was used to enhance the vessel structure in Figure 1e. The subsequent figures provide images of skeletal elements (Figure 2 and Movie S1, S2), organ structures (Figure 3 and Movie S3) and vasculature (Figure 4, 5 and Movie S4, S5). Using volume rendering software, three-dimensional representations of anatomical structures were constructed from cross-sectional images, thereby creating digital models movable in space (Movie S1–S5).

Both CT and MRI were capable to generate visualisations of soft tissues (Figure 3). The use of an ingested MRI contrast agent in the white knee tarantula increased the detectable signal from the gastrointestinal tract (Figure 3c, 3d and Movie S3–S5). Irrespective of the smaller sensibility of the CT modality to soft tissue structures, visualisation of major organ structures was still possible, indeed after vascular filling with a contrast agent, exemplified in Figure 3a–b, where the heart and the closely aligned lungs and liver of the alligator are easily distinguished. In cases where adjacent tissues exhibit big differences in radio-absorbance, e.g. air and tissue in lungs and trachea, high-resolution representations of borderline areas can be produced using CT (Figure 1f, 3a, 3b).

The intravenous use of contrast agents, such as gadolinium-containing agents for MRI and lead oxide or barium sulphate for CT in solidifying solutions, made it possible to produce ex vivo angiographies (Figure 4, 5 and Movie S4, S5). Perfusion of the cane toad's lungs with barium sulphate prior to CT allowed visualisation of the pulmonary vessels (Figure 4b). Post-processing and analysis allow for at digital dissection of the animal, thereby isolating the cane toad's lungs from the surrounding tissue (Figure 4c). Whole body angiography can be performed with a similar method, exemplified with the yellow anaconda and the domestic pig (Figure 4d, 5a and Movie S4, S5). Additionally, by preparing the contrast agent to a suitable viscosity, making it impenetrable to the capillary barrier, either the arterial or the venous side of the vasculature can be visualised separately as exemplified by the MRI venography of the red-eared slider (Figure 1c). High-resolution μCT imaging allows for detection of minute vessel structures, facilitating a complete representation of the vasculature in the head region of the air-breathing rice field eel (Figure 4a). The use of vascular contrast agents and μCT imaging revealed the microvasculature of the coronary arteries in the heart of the yellow anaconda (Figure 5c). Coronary arteriography of a giraffe heart (Figure 5b), as well as a whole body angiography of the domestic pig (Figure 5a and Movie S5), were conducted to display the mammalian vasculature.

## Discussion

This methodological study demonstrates that MRI and CT with appropriate post-processing methods can provide anatomical descriptions of various structures in animals in quality comparable with traditional dissection techniques [18]–[20], [30]–[33]. Main skeletal architecture was easily visualised (Figure 2 and Movie S1, S2), and it was possible to identify the structure and position of major visceral organs, such as heart, liver, lungs and gastrointestinal tract (Figure 1d, Figure 3 and Movie S3). The vasculature could be visualised using solidifying contrast agents (Figure 1d, 1e, 4, 5 and Movie S4, S5). Further, we demonstrated the ability of μCT to acquire images with very high spatial resolution, exemplified by visualisation of the rice field eel vasculature (Figure 4a) and the coronary microvasculature of the anaconda heart (Figure 5c).

Both MRI and CT are non-invasive techniques, leaving the animal intact, except in situations where angiographic procedures require vascular filling with a solidifying contrast agent. In fact, because both modalities can be used on live animals, MRI and CT allow for repeated measures on the same individual, which call for longitudinal investigations of anatomical phenomena, e.g. investigations of tissue regeneration and volumetric changes in the digestive system during digestion [34]. Note, however, that both CT and MRI require that the animal is completely immobilized during the entire scan procedure, which necessitates respiratory or heart-beat triggering to avoid most artefacts.

MRI and CT complement each other well [35], where MRI is useful to reveal soft tissue structures with subtle differences in composition and can provide images from various angles, and multi-slice CT can produce excellent images of hard calcified structures. The two techniques differ considerably in the speed at which the images can be obtained. While a high-resolution CT acquisition can be performed in less than a minute, a high-resolution MRI usually requires up to hours depending on inherent sequence parameters, magnet field strength, etc. Yet, this technique is relatively fast compared to traditional preservation and dissection procedures. Modern clinical MRI and CT modalities are able to generate images with a maximum resolution of approximately 0.09–0.25 mm^2^ with an acceptable signal-to-noise ratio. Introduction of experimental high-field MRI or μCT is a way to increase the spatial resolution, allowing MRI image resolution in the order 1000–3000 µm^2^ [36] and μCT images down to a voxel size of 1–10 µm^2^. In the present study, μCT was used to reveal minute structures of the rice field eel vasculature (Figure 4a) and coronary architecture of arteries in the yellow anaconda heart (Figure 5c). These delicate structures are prone to collapse and disintegrate during a thorough dissection under the microscope. The same is true for lung tissue in the turtle (Figure 1f) and toad (Figure 4b, c) as well as the gastrointestinal tract in the tarantula (Figure 3c, d).

As discussed by Ziegler et al. [37], a challenge us the enormous amount of data generated, and there is a current need for dedicated databases, where data are stored in a standardized fashion. Besides whole animal reproductions, detailed reproductions of specific organs can be derived from segmentation and manual cropping (Figure 3b and 4c). The development in processing algorithms is likely to facilitate automatic segmentation of entire animals based on geometric definitions of vascular, skeletal and organ structures. However, such automatic process requires specialized information about specific anatomical structures of each species of interest in order to delineate anatomical structures. If such laborious step is feasible, we hypothesise that future anatomical visualisations could be processed relatively fast due to the swiftness in modern electronic data processing.

An important benefit of performing dissection on a computerised digital model compared to a real specimen is the forgiveness of this technique. A wrong cut does not wreck a whole, possibly rare, specimen. Digitalized catalogues of museum's type specimens would allow for various investigations of anatomical characters and may increase the accessibility of phenotypical data, exemplified by Ziegler and colleagues [22]–[24] who include museum specimens in their morphological research on echinoderms, as well as the impressive catalogue of skeletal and especially cranial morphology in the animal kingdom presented by the Digital Morphology library [27]. Visualisations of inflated lungs (Figure 1f, 3a, 3b and 4b, 4c) are examples of structures that would normally collapse during dissection due to the change in intrathoracic pressure, and thus cause a major dissection artefact. However, this is not the case with non-invasive CT, where the thorax is left unopened and no geometric artefacts appear. Traditional dissections remain necessary for many purposes, but digital pre-dissection with MRI and CT could improve the planning of the dissection as well as contribute with valuable additional knowledge.

In conclusion, given that clinical scanners found in the majority of larger hospitals are fully suitable for these purposes, we encourage biologists to take advantage of these imaging techniques in creation of three-dimensional graphical representations of internal structures.

## Supporting Information

Movie S1 **CT movie of red-eared slider (** ***Trachemys scripta*** **).** Initially the animal is turned 360° along its long axis with a slight change in contrast settings halfway, subsequently the plastron is digitally dissected away to reveal internal skeletal architecture.
(MP4)

Movie S2 **Interactive CT movie of African Savannah monitor (** ***Varanus exanthematicus*** **).** (Opened in QuickTime Player it is possible to rotate the three dimensional model).
(MOV)

Movie S3 **MRI movie of whiteknee tarantula (** ***Acanthoscurria geniculate*** **).** Initially the animal is turned 360° along its long axis, then contrast settings are changed to reveal the contrast agent filled gastrointestinal tract.
(MP4)

Movie S4 **CT movie of the vasculature of a yellow anaconda (** ***Eunectes notaeus*** **).** Initially contrast settings are changed slightly to present only vertebrae and contrast agent filled vasculature, followed by a quick survey of the entire snake's body.
(MP4)

Movie S5 **CT movie of the vasculature of a domestic pig (** ***Sus scrofa domesticus*** **).** Initially the animal is turned 360° along its long axis, then contrast settings are changed to reveal the contrast filled vasculature, followed by a quick survey of the pig body.
(MP4)
